# Supplementary material for: Reconciling Mining with the Conservation of Cave Biodiversity: A Quantitative Baseline to Help Establish Conservation Priorities
Source: PLoS One. 2016 Dec 20;11(12):e0168348. doi: 10.1371/journal.pone.0168348 (PMC5173368; doi:10.1371/journal.pone.0168348)
Supplement: S1 Dataset — (ZIP) [file pone.0168348.s002.zip › Taxa/Serra Norte/SN_2013/N4E/Lista N4E-60.pdf]

CAVIDADE N4E-0060

Classe	Ordem	Fam/Outros	Gên/Outros	Espécie	S-U	U-S	Zon.
Arachnida	Araneae	Filistatidae		jovem	X		E
Arachnida	Araneae	Pholcidae	*Leptopholcus*	sp.1		X	E
Arachnida	Araneae	Pholcidae	*Mesabolivar*	sp.1	X	X	E
Arachnida	Araneae	Scytodidae	*Scytodes*	*eleonorae*	2		E
Arachnida	Araneae	Theridiosomatidae	*Plato*	sp.1	X	X	E
Entognatha	Collembola	Paronellidae		sp.1		X	E
Insecta	Coleoptera			jovem	X	X	E
Insecta	Diptera	Aulacigastridae		sp.		X	E
Insecta	Diptera	Cecidomyiidae	Cecidomyiinae	sp.	X		E
Insecta	Diptera	Dolichopodidae		sp.	X	X	E
Insecta	Heteroptera	Reduviidae	Emesinae	sp.7	1		E
Insecta	Homoptera	Cicadellidae		sp.2		X	E
Insecta	Hymenoptera	Formicidae	*Camponotus*	sp.1		X	E
Insecta	Hymenoptera	Formicidae	*Camponotus*	sp.2	X		E
Insecta	Isoptera	Termitidae	*Nasutitermes*	sp.	X	X	E
Insecta	Lepidoptera			jovem		X	E
Insecta	Orthoptera	Phalangopsidae	*Paraclodes*	sp.	1	2	E
Insecta	Psocoptera			jovem	X		E
Mammalia	Chiroptera	Emballonuridae	*Peropteryx*	*kappleri*	1	1	E
